# A case of malignant pheochromocytoma with neurofibromatosis type 1 having difficulty in differentiating spinal tumor

**DOI:** 10.1002/iju5.12751

**Published:** 2024-06-09

**Authors:** Kohei Segawa, Yoshiyuki Yamamoto, Taigo Kato, Koji Hatano, Yoichi Kakuta, Atsunari Kawashima, Shinichiro Fukuhara, Norio Nonomura

**Affiliations:** ^1^ Department of Urology Osaka University Graduate School of Medicine Suita Osaka Japan

**Keywords:** CVD, malignant pheochromocytoma, neurofibromatosis type 1, spinal tumor

## Abstract

**Introduction:**

Neurofibromatosis type 1 is a hereditary condition often associated with pheochromocytomas but rarely with malignant pheochromocytomas. Neurofibromatosis type 1 is often associated with bone lesions, which complicates the distinction between malignant and benign tumors.

**Case presentation:**

A 46‐year‐old man with a medical history of neurofibromatosis type 1 presented with right abdominal pain. Computed tomography revealed a right adrenal tumor, and metaiodobenzylguanidine scintigraphy showed accumulation in the right adrenal gland and thoracic vertebrae. He was diagnosed with pheochromocytoma, and a right adrenalectomy was performed. After surgery, a bone biopsy was conducted on the spinal lesion, confirming metastasis of pheochromocytoma, prompting irradiation. After that, lung and liver metastases emerged, and chemotherapy with cyclophosphamide, vincristine, and dacarbazine was initiated; however, the disease progressed, and he died 11 months after surgery.

**Conclusion:**

We report a case of malignant pheochromocytoma associated with neurofibromatosis type 1 in which bone metastasis was difficult to diagnose.

**Abbreviations & Acronyms**
CT: computed tomography
CVD: cyclophosphamide, vincristine, and dacarbazine
FDG‐PET: (^18^F)‐2‐fluoro‐2‐deoxy‐D‐glucose‐positron emission tomography
H&E: hematoxylin–eosin stain
MIBG: metaiodobenzylguanidine
MRI: magnetic resonance imaging
NF1: neurofibromatosis type 1
SUVmax: maximum of standardized uptake value

**Keynote message**

Here, we report a case of malignant pheochromocytoma with neurofibromatosis type 1, manifesting with spinal metastases. As neurofibromatosis type 1 has a high rate of benign spinal cord lesions, it is important to distinguish it from bone metastases in cases of malignant complications.

## Introduction

NF1 is the most prevalent autosomal‐dominant inherited disorder and is associated with various benign and malignant tumors.^1^, ^2^ Pheochromocytomas occur more frequently in patients with NF1 than in the general population,^3^ with malignant occurrences being rare.

The 5‐year overall survival rate for malignant pheochromocytomas is 55–75%, signifying a generally poor prognosis.^4^, ^5^ The frequency of bone metastasis is high in patients with malignant pheochromocytoma.^5^, ^6^ Some spinal tumors, including neurofibromatosis and schwannoma, are relatively common in 13–65% of patients with NF1.^1^, ^7^, ^8^ It can be difficult to distinguish spinal tumors with NF1 from other malignant bone tumors, including metastasis. This case report describes a patient with malignant pheochromocytoma associated with NF1 who presented with thoracic vertebral metastases.

## Case presentation

A 46‐year‐old male visited a physician with complaints of right‐sided abdominal pain. Right adrenal pheochromocytoma and multiple bone metastases were suspected, prompting a visit to our hospital. He had a history of NF1 and a compression fracture of the thoracic vertebrae, possibly due to a spinal tumor. At the time of visit, he was taking 8 mg of doxazosin, and his blood pressure was normal without any symptoms. Numerous café‐au‐lait spots and neurofibromas were observed on the left forearm and back. Levels of noradrenaline and dopamine in the blood, and metanephrines and normetanephrine in 24‐h urine storage tests were high (Table S1).

**Fig. 1:**
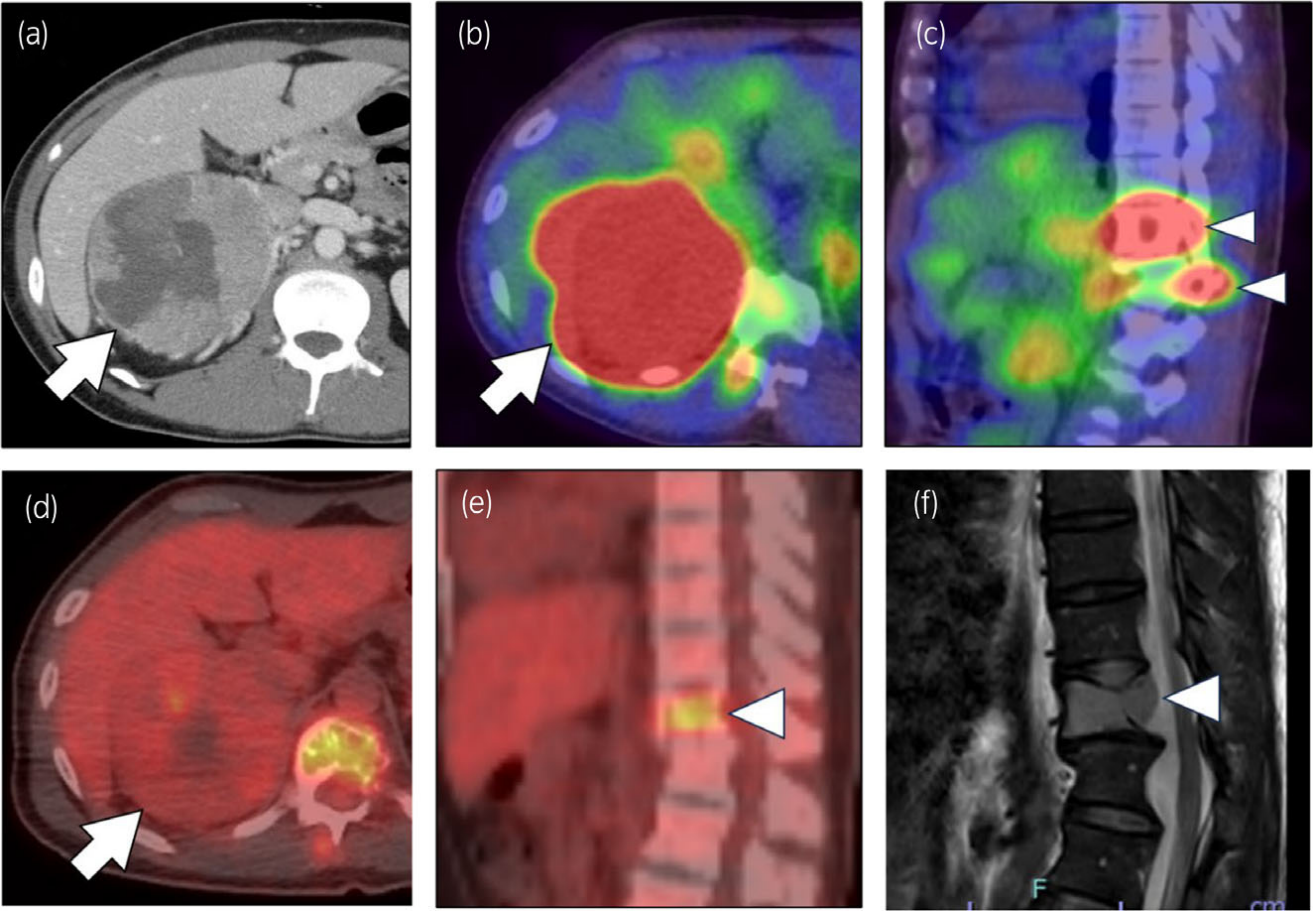
(a) Abdominal contrast CT shows a right adrenal mass (arrow) of 93 mm in size with internal hemorrhage and necrosis. (b, c) 123I‐MIBG scintigraphy shows multiple accumulations in the right adrenal mass (arrow) (b), 11th thoracic vertebral body, and 12th thoracic vertebral arch (arrowheads) (c). (d, e) FDG‐PET CT scan shows accumulation in the adrenal mass (SUVmax 4.0) (arrow) and thoracic vertebra (SUVmax 5.3) (arrowhead). (f) MRI T2‐weighted sagittal image shows loss of normal fatty marrow and high signal in the 11th thoracic vertebra (Arrowhead), with dural sac dilatation at the level of the 11th and 12th thoracic vertebra.

Abdominal contrast CT revealed a right adrenal mass, 93 mm in size (Fig. 1a). 123I‐MIBG scintigraphy also showed multiple accumulations in the right adrenal mass, 11th thoracic vertebral body, and 12th thoracic vertebral arch (Fig. 1b,c) and weak abnormal accumulation in the right iliac bone. FDG‐PET showed accumulation in the adrenal mass (SUVmax 4.0) (Fig. 1d) and the 11th thoracic vertebra (SUVmax 5.3) (Fig. 1e). MRI showed the spinal cord tumor was accompanied by dural sac extension, suggesting a schwannoma of NF1 origin (Fig. 1f). Using imaging alone, it was difficult to differentiate whether the spinal tumor was metastatic or a neurogenic tumor of NF1 origin.

**Fig. 2:**
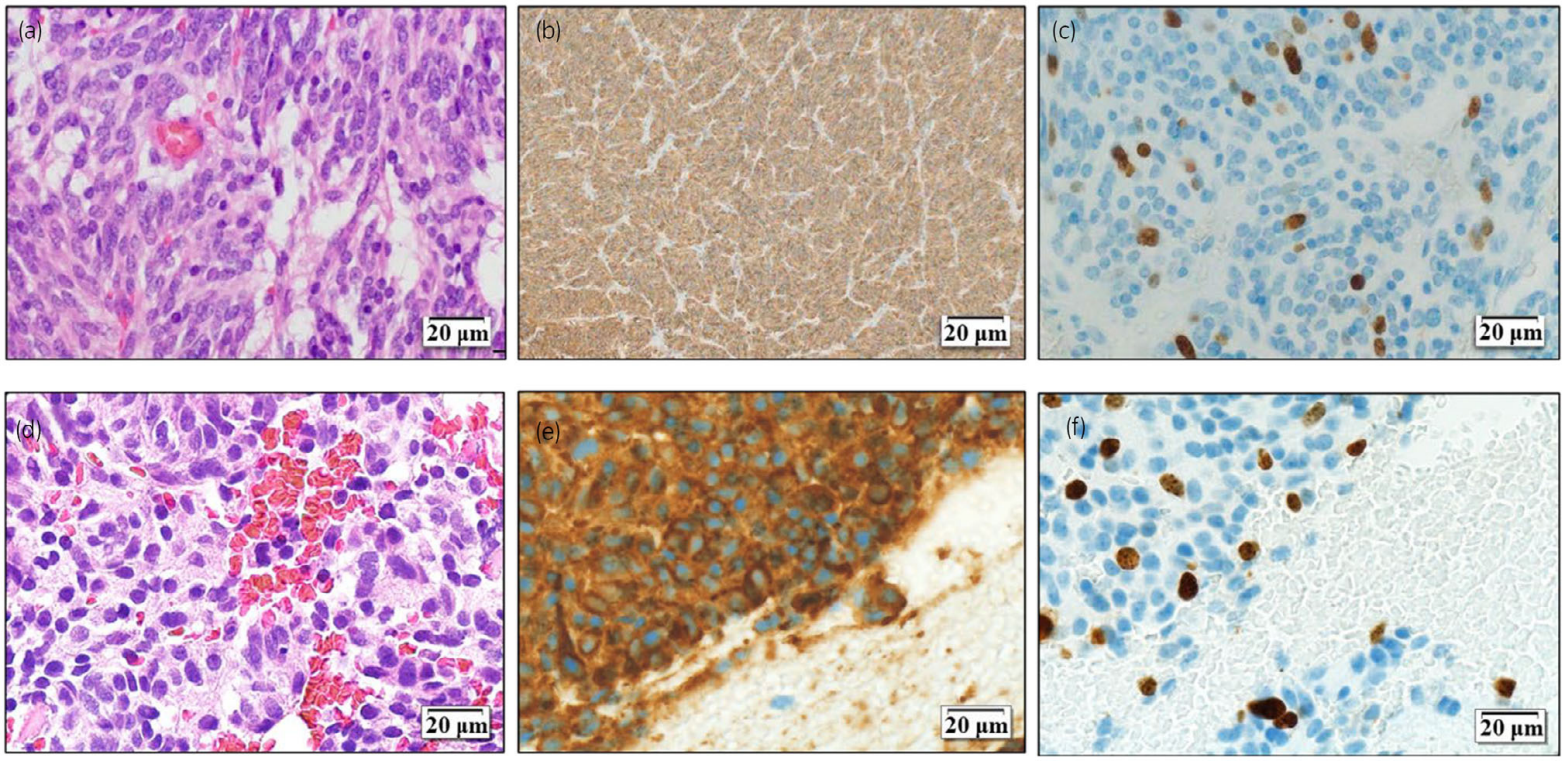
(a–c) A pathological specimen after total adrenalectomy (H&E (a), chromogranin A (b), and Ki67 staining (c), respectively), positive for chromogranin A, suggesting pheochromocytoma and Ki‐67 labeling index was 8.8%. (d–f) A pathological specimen of CT‐guided needle biopsy of a spinal tumor (H&E (d), chromogranin A (e), and Ki67 staining (f), respectively) was positive for chromogranin A, suggesting metastasis of pheochromocytoma and Ki‐67 labeling index was 10%.

Although a spinal biopsy was necessary to confirm the diagnosis, we opted for adrenalectomy due to the potential risk of a hypertensive crisis and for tumor reduction. The operation was initiated in the supine position, and a chevron incision was placed. During the open right adrenalectomy, minor adhesions of the tumor to surrounding tissues resulted in a surgery time of 5 h 32 min, with a blood loss of 320 mL. Pathology was mostly positive for chromogranin A, leading to a diagnosis of pheochromocytoma (Fig. 2a–c). The Ki‐67 labeling index was 8.8%, and the Pheochromocytoma of the Adrenal gland Scaled Score (PASS)^9^ was 4 points, indicating malignancy (Fig. 2a–c). The Grading System for Adrenal Pheochromocytoma and Paraganglioma^10^ score was 5 points, indicating moderately differentiated type. Pathological margins were negative.

**Fig. 3:**
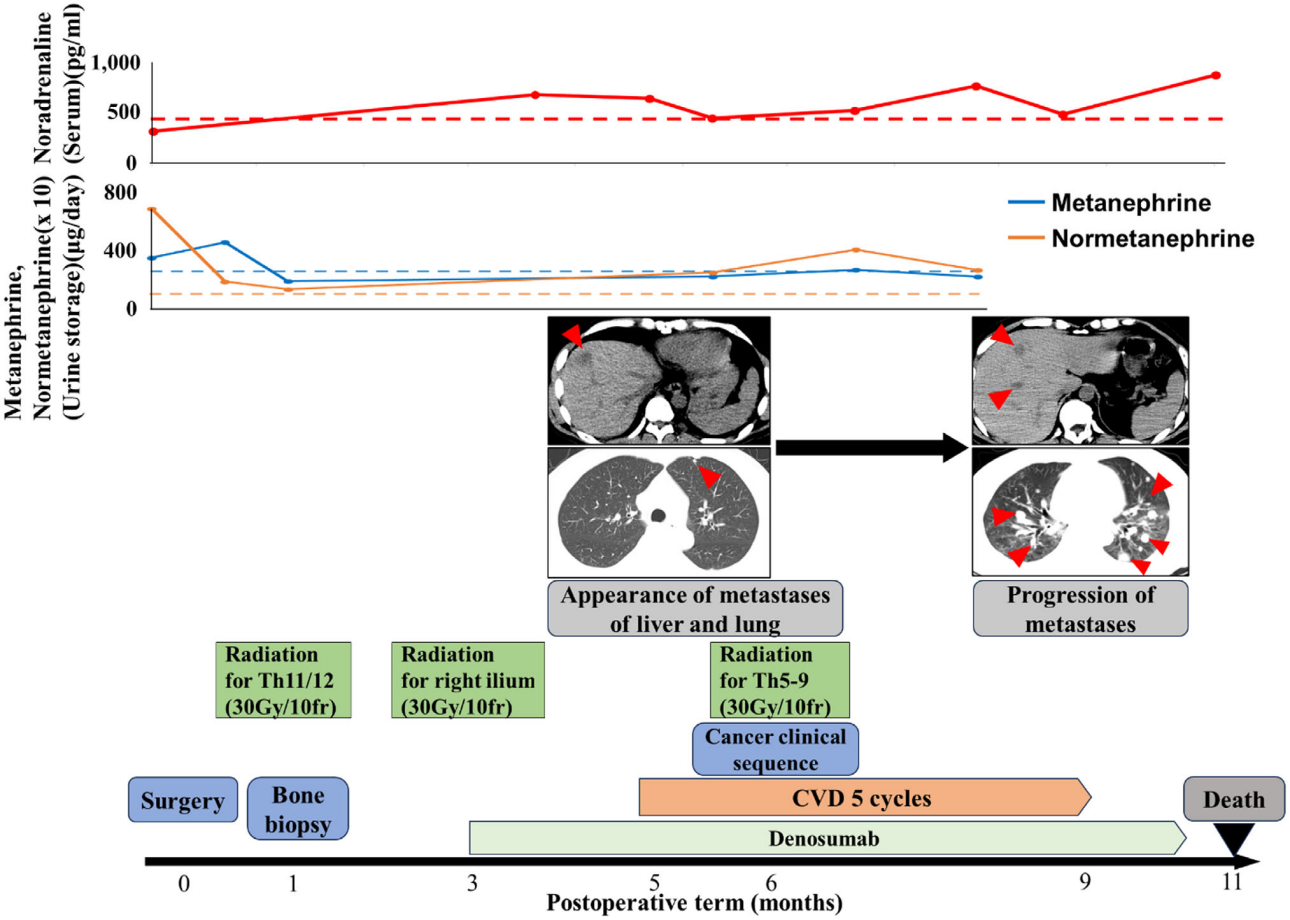
Clinical course after right adrenalectomy. Arrowheads were metastases. The dotted line was the upper limit of normal levels for serum noradrenaline, urinary metanephrine, and normetanephrine. Urinary metanephrine and normetanephrine were measured by 24‐h urine storage test.

**Fig. 4:**
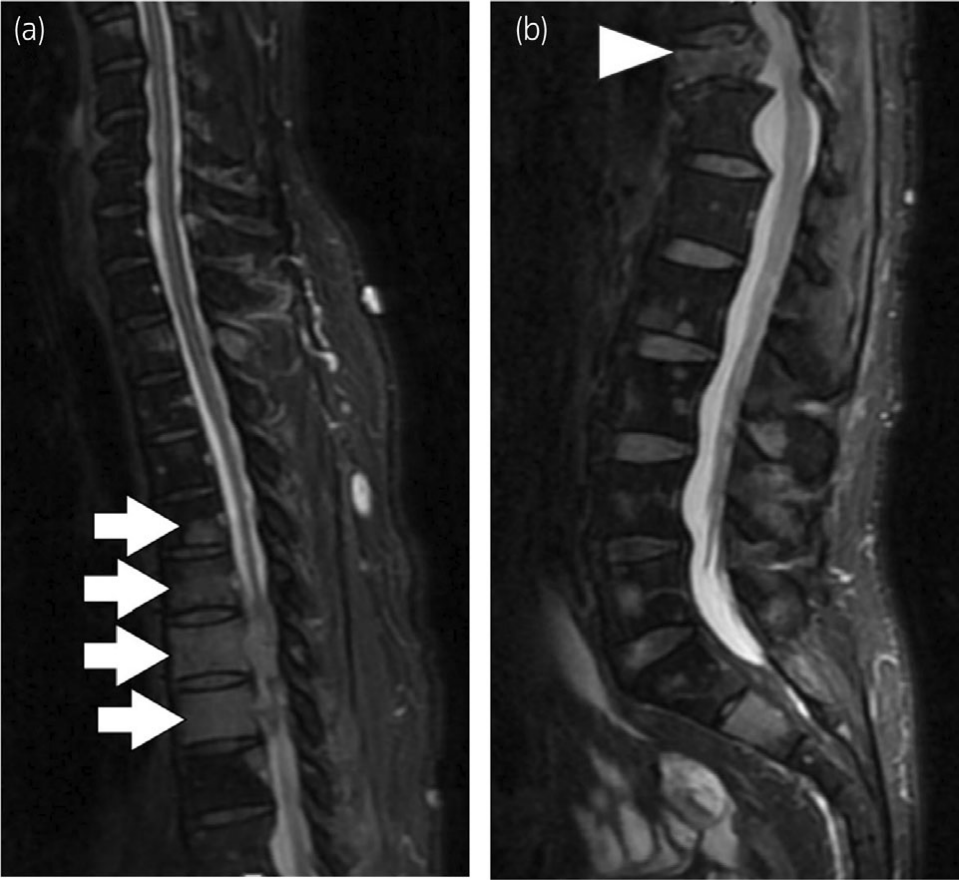
MRI of a patient with onset of lower extremity muscle weakness. Fat‐suppressed T2‐weighted image showing multiple high‐signal areas in the spine. (a) It extends into the spinal canal in the 5th–8th thoracic vertebrae (arrows). (b) A known fracture of the vertebral body in the 11th thoracic vertebra (arrowhead).

One month postoperatively, a CT‐guided needle biopsy confirmed pheochromocytoma metastasis in the 11th thoracic spine lesion (Fig. 2d–f). He continued taking doxazosin, and there were no complications associated with the biopsy. The patient's clinical course is shown in Figure 3. Radiation (30 Gy/10 fr) was administered to the 11th–12th thoracic spine metastases. Radionuclide therapy was not covered by insurance at the time of treatment, and the patient did not request it. Three months after surgery, the patient was diagnosed with bone metastasis due to an increase in the right iliac lesion and was treated with both irradiation (30 Gy/10 fr) and denosumab. Five months after surgery, liver and lung metastases appeared, and chemotherapy with CVD was administered. Six months after surgery, muscle weakness in the lower limbs developed, and MRI indicated worsening spinal metastasis (Fig. 4a,b). Additional irradiation (30 Gy/10 fr) was performed on the 5th–9th thoracic vertebral lesions. The level of serum noradrenaline and urinary normetanephrine did not become negative after surgery and tended to increase as the disease progressed (Fig. 3). Cancer clinical sequence testing with the OncoGuide™ NCC Oncopanel System (Sysmex Corporation, Kobe, Japan) was performed. However, neither somatic nor germline mutations, including NF1, were detected. Five courses of CVD were administered, yet the disease progressed, and the patient died 11 months after surgery.

## Discussion

NF1 is an autosomal‐dominant disorder characterized by skin lesions like neurofibromas and café‐au‐lait spots and various bone, eye, and nervous system manifestations.^1^ Although we could not detect NF1 mutations in this case, next‐generation sequencing could not detect mutations in 7.0% of NF1 cases in the previous report.^11^ NF1 is a relatively common genetic disorder, occurring in 1 in about 2600‐4950 births worldwide.^2^, ^12^ Various benign and malignant tumors occur, with a high rate of neurogenic tumors.^1^ Pheochromocytomas manifest in 0.1%–5.7% of patients with NF1,^3^ with a rate of malignant transformation comparable to general malignant pheochromocytomas.^1^, ^3^ A previous report detailed 10 cases of metastatic pheochromocytoma associated with NF1, with a median age of 46 years and median tumor size of 6 cm.^13^ The most common site of metastasis was the bone (50%), followed by the lung and liver,^13^ as with general malignant pheochromocytoma.^6^

NF1 is often associated with bone involvement, which is important in differentiating it from bone metastasis. Patients with NF1 have a relatively high frequency of spinal tumors.^1^, ^7^, ^8^ With respect to histology, most spinal tumors with NF1 are neurofibromas, rarely schwannomas.^14^ Malignant peripheral schwannomas affect 4.6% of patients with NF1, 55% of which involve the trunk.^15^

In this case, a compression fracture due to a spinal tumor was observed before diagnosing pheochromocytoma, and schwannoma with NF1 was first suspected based on imaging findings, as dural sac extension was observed on MRI. MIBG scintigraphy revealed abnormal accumulation, which increased the possibility of pheochromocytoma metastasis. Nevertheless, MIBG, including 123I and 131I, is not sensitive enough for malignant pheochromocytoma, and Timmers reported a sensitivity of 65% in SDHB mutation‐positive cases.^16^ Reports exist of 123I‐MIBG accumulation in juxta‐adrenal schwannoma.^17^ Therefore, the possibility that the spinal tumor was also a schwannoma in this case could not be excluded.

PET‐CT may be useful in localizing metastatic pheochromocytoma.^18^ Previous review showed significant differences in mean SUVmax between benign neurofibromas and malignant peripheral schwannomas in patients with NF1, and the general cut‐off value of SUVmax was 3–4.^19^ In this case, the bone SUVmax value of the supine lesion was 5.3, and distinguishing between metastatic or benign tumors remained challenging.

In conclusion, we report a rare case of NF1‐associated malignant pheochromocytoma with bone metastasis. In this case, it was difficult to diagnose whether the spinal tumor originated from NF1 or was a pheochromocytoma metastasis. Malignant pheochromocytomas have a poor prognosis; hence, prompt diagnosis of metastases is important.

## Author contributions

Kohei Segawa: Conceptualization; data curation; investigation; writing – original draft. Yoshiyuki Yamamoto: Conceptualization; investigation; project administration; writing – original draft. Taigo Kato: Supervision; writing – review and editing. Koji Hatano: Supervision; writing – review and editing. Yoichi Kakuta: Supervision; writing – review and editing. Atsunari Kawashima: Supervision; writing – review and editing. Shinichiro Fukuhara: Supervision; writing – review and editing. Norio Nonomura: Project administration; supervision; writing – review and editing.

## Conflict of interest

The authors declare no conflict of interest.

## Approval of the research protocol by an Institutional Reviewer Board

Not applicable.

## Informed consent

Written informed consent was obtained from the patient to publish this case report and accompanying images.

## Registry and the Registration No. of the study/trial

Not applicable.

## Supporting information


**Table S1.** Pretreatment adrenal medullary hormone levels in blood and urine.

## References

[1] Gutmann DH , Ferner RE , Listernick RH , Korf BR , Wolters PL , Johnson KJ . Neurofibromatosis type 1. Nat. Rev. Dis. Primers 2017; 3: 17004. 28230061 10.1038/nrdp.2017.4

[2] Huson SM , Compston DA , Clark P , Harper PS . A genetic study of von Recklinghausen neurofibromatosis in south east Wales. I. Prevalence, fitness, mutation rate, and effect of parental transmission on severity. J. Med. Genet. 1989; 26: 704–711. 2511318 10.1136/jmg.26.11.704 PMC1015740

[3] Walther MM , Herring J , Enquist E , Keiser HR , Linehan WM . von Recklinghausen's disease and pheochromocytomas. J. Urol. 1999; 162: 1582–1586. 10524872

[4] Amar L , Baudin E , Burnichon N *et al*. Succinate dehydrogenase B gene mutations predict survival in patients with malignant pheochromocytomas or paragangliomas. J. Clin. Endocrinol. Metab. 2007; 92: 3822–3828. 17652212 10.1210/jc.2007-0709

[5] Choi YM , Sung TY , Kim WG *et al*. Clinical course and prognostic factors in patients with malignant pheochromocytoma and paraganglioma: a single institution experience. J. Surg. Oncol. 2015; 112: 815–821. 26464058 10.1002/jso.24063

[6] Hamidi O , Young WF Jr , Iñiguez‐Ariza NM *et al*. Malignant pheochromocytoma and paraganglioma: 272 patients over 55 years. J. Clin. Endocrinol. Metab. 2017; 102: 3296–3305. 28605453 10.1210/jc.2017-00992 PMC5587061

[7] Sial M , George KJ . A review of spinal lesions in neurofibromatosis type 1 in a large neurofibromatosis type 1 center. World Neurosurg. 2023; 169: e157–e163. 36334707 10.1016/j.wneu.2022.10.100

[8] Thakkar SD , Feigen U , Mautner VF . Spinal tumours in neurofibromatosis type 1: an MRI study of frequency, multiplicity and variety. Neuroradiology 1999; 41: 625–629. 10525761 10.1007/s002340050814

[9] Thompson LD . Pheochromocytoma of the Adrenal gland Scaled Score (PASS) to separate benign from malignant neoplasms: a clinicopathologic and immunophenotypic study of 100 cases. Am. J. Surg. Pathol. 2002; 26: 551–566. 11979086 10.1097/00000478-200205000-00002

[10] Kimura N , Takayanagi R , Takizawa N *et al*. Pathological grading for predicting metastasis in phaeochromocytoma and paraganglioma. Endocr. Relat. Cancer 2014; 21: 405–414. 24521857 10.1530/ERC-13-0494

[11] Maruoka R , Takenouchi T , Torii C *et al*. The use of next‐generation sequencing in molecular diagnosis of neurofibromatosis type 1: a validation study. Genet. Test. Mol. Biomarkers 2014; 18: 722–735. 25325900 10.1089/gtmb.2014.0109 PMC4216997

[12] Lammert M , Friedman JM , Kluwe L , Mautner VF . Prevalence of neurofibromatosis 1 in German children at elementary school enrollment. Arch. Dermatol. 2005; 141: 71–74. 15655144 10.1001/archderm.141.1.71

[13] Kumar S , Lila AR , Memon SS *et al*. Metastatic cluster 2‐related pheochromocytoma/paraganglioma: a single‐center experience and systematic review. Endocr. Connect. 2021; 10: 1463–1476. 34662294 10.1530/EC-21-0455 PMC8630763

[14] Safaee M , Parsa AT , Barbaro NM *et al*. Association of tumor location, extent of resection, and neurofibromatosis status with clinical outcomes for 221 spinal nerve sheath tumors. Neurosurg. Focus. 2015; 39: E5. 10.3171/2015.5.FOCUS15183 26235022

[15] Ducatman BS , Scheithauer BW , Piepgras DG , Reiman HM , Ilstrup DM . Malignant peripheral nerve sheath tumors. A clinicopathologic study of 120 cases. Cancer 1986; 57: 2006–2021. 3082508 10.1002/1097-0142(19860515)57:10<2006::aid-cncr2820571022>3.0.co;2-6

[16] Timmers HJ , Kozupa A , Chen CC *et al*. Superiority of fluorodeoxyglucose positron emission tomography to other functional imaging techniques in the evaluation of metastatic SDHB‐associated pheochromocytoma and paraganglioma. J. Clin. Oncol. 2007; 25: 2262–2269. 17538171 10.1200/JCO.2006.09.6297

[17] Tommaselli AP , Valentino R , Rossi R *et al*. Usefulness of 123I‐metaiodobenzylguanidine (MIBG) scintiscan in the diagnosis of juxta‐adrenal schwannoma. J. Clin. Endocrinol. Metab. 1996; 81: 843–846. 8636313 10.1210/jcem.81.2.8636313

[18] Feng B , Chen M , Jiang Y , Hui Y , Zhao Q . ^18^F‐FDG PET/CT in a patient with malignant pheochromocytoma recurrence and bone metastasis after operation‐case report and review of the literature. Front. Med. 2021; 8: 733553. 10.3389/fmed.2021.733553 PMC8633843 34869428

[19] Tovmassian D , Abdul Razak M , London K . The role of [^18^F]FDG‐PET/CT in predicting malignant transformation of plexiform neurofibromas in neurofibromatosis‐1. Int. J. Surg. Oncol. 2016; 2016: 6162182. 28058117 10.1155/2016/6162182 PMC5183794

