# Supplementary material for: A case of malignant pheochromocytoma with neurofibromatosis type 1 having difficulty in differentiating spinal tumor
Source: IJU Case Rep. 2024 Jun 9;7(4):336–40. doi: 10.1002/iju5.12751 (PMC11221939; doi:10.1002/iju5.12751)
Supplement: Supplementary file 1 — Table S1. Pretreatment adrenal medullary hormone levels in blood and urine. [file IJU5-7-336-s001.docx]

**Supplemental table 1 Pre-treatment adrenal medullary hormone levels in blood and urine**

| **Blood test** |  |  | Normal range |  |
| --- | --- | --- | --- | --- |
| Adrenaline (pg/ml) | 52 | ( | 0 - 100 | ) |
| Noradrenaline (pg/ml) | 1,159 | ( | 100 - 450 | ) |
| Dopamine (pg/ml) | 26 | ( | 0 - 20 | ) |
| **24-hour urine storage test** |  |  |  |  |
| Metanephrine (mg/day) | 0.34 | ( | 0.04 - 0.20 | ) |
| Normetanephrine (mg/day) | 7.2 | ( | 0.09 - 0.28 | ) |
| Vanillylmandelic acid (mg/day) | 19.9 | ( | 1.4 - 4.9 | ) |
